# Oncofertility awareness among primary care physicians in India

**DOI:** 10.12688/f1000research.126232.2

**Published:** 2023-09-28

**Authors:** Prathima Tholeti, Shubhashree Uppangala, Rajesh Kumar Jayaram, Karthik S Udupa, Guruprasad Kalthur, Norah Spears, Teresa Woodruff, Satish K Adiga

**Affiliations:** 1 Centre for Fertility Preservation, Division of Clinical Embryology, Department of Reproductive Science, Kasturba Medical College, Manipal, Manipal Academy of Higher Education, Manipal, 576 104, India; 2 Division of Reproductive Genetics, Department of Reproductive Science, Kasturba Medical College, Manipal, Manipal Academy of Higher Education, Manipal, 576 104, India; 3 Vijay Hospital, Hosur, Tamil Nadu, 635109, India; 4 Department of Medical Oncology, Kasturba Medical College, Manipal, Manipal Academy of Higher Education, Manipal, 576 104, India; 5 Division of Reproductive Biology, Department of Reproductive Science, Kasturba Medical College, Manipal, Manipal Academy of Higher Education, Manipal, 576 104, India; 6 Department of Biomedical Sciences, University of Edinburgh, Edinburgh, EH8 9XD, UK; 7 Oncofertility Consortium, Room A626B, Michigan State University, East Lansing, Michigan, 48824-1316, USA

**Keywords:** Fertility preservation, oncofertility, primary care physicians, general physicians, paediatricians

## Abstract

Background:

Primary care physicians not only coordinate referrals to oncology services but can play a crucial role in successful fertility preservation referrals in cancer-diagnosed patients. Hence, it is important to assess their knowledge and attitudes towards fertility preservation.

Methods:

An eighteen-item oncofertility survey was administered to primary care physicians between May 2019 to September 2020.

Results:

A total of forty-six responses were received and analysed . About 60% of primary care physicians did not have adequate knowledge about available fertility preservation options and only 26-32% were aware of international guidelines recommending fertility preservation in cancer patients.

Conclusions:

Imparting awareness and knowledge of fertility preservation and its options to primary care physicians could enable an integrated cancer care model while also facilitating successful oncofertility referrals in countries like India.

## Introduction

Oncofertility – integrating cancer care with fertility preservation (FP) – is expected to be an essential part of the standard care for patients, ^1^ with international guidelines recommending fertility preservation counselling as routine for reproductive-age cancer patients and for prepubertal children undergoing gonadotoxic therapies. ^2^ However, successful implementation of oncofertility care requires a coordinated referral system among healthcare providers. ^3^ While it is established that the role of oncologists and reproductive specialists is key for successful oncofertility, ^3^^,^^4^ it is also believed that primary care physicians (PCPs) can play an important role in ensuring successful fertility preservation referrals. ^5^

PCPs inclusive of general physicians (GPs) and paediatricians (PEDs), are considered as gatekeepers of the healthcare system who provide comprehensive, individual-centric and coordinated continuous care. ^6^ PCPs are the initial contact point for most cancer patients, who are then referred to specialists such as medical oncologists or paediatric hemato-oncologists. ^7^ However, the role of PCPs in cancer care has expanded further across the cancer care continuum from prevention, diagnosis, treatment to cancer survivorship as it has been reported that cancer patients preferred their PCPs to be involved in their cancer care. ^7^^,^^8^ A recent study has reported patients discussing their cancer treatment options with their PCPs, in addition to their oncologists, demonstrating the important role they play in decision-making. ^8^ It has been reported that about two thirds of patients with long-term or complex health issues prefer interpersonal continuity with a PCP whom they know, trust and who also knows their medical history. ^9^^,^^10^ Hence, PCPs not only coordinate referrals, but are also involved in providing general medical care, emotional support and overall decision making for the patient during cancer care. ^11^ The American Society of Clinical Oncology breast cancer survivorship guidelines, recommend post-treatment physical and psychosocial surveillance by primary care clinicians and expediting referrals to reproductive endocrinologists in survivors experiencing infertility. ^12^

Fertility preservation while at times is considered to be a burden for patients and their families, can provide opportunities for maximizing reproductive potential of the patient. A multi-disciplinary team that includes the patient’s primary care physician along with the cancer specialists and reproductive specialists will allow for a comprehensive decision-making by the patients. ^13^ This also allows for families to gather the information required for taking informed decisions regarding fertility preservation thereby maximizing the chances for preserving their fertility before initiation of cancer therapy. ^13^ Similar approaches are applicable to patients with non-oncological conditions requiring gonadotoxic treatments. ^13^ It is therefore important for PCPs dealing with cancer patients or patients with non-malignant conditions requiring gonadotoxic therapies, to be knowledgeable both of the adverse gonadotoxic effects of chemo- and radiotherapy as well as the options available to mitigate them. The awareness of PCPs of international fertility preservation guidelines and on the available fertility preservation options are expected to strengthen the likelihood of fertility preservation referrals. ^11^ Therefore, this study was aimed to assess the awareness, knowledge and perceptions of PCPs towards fertility preservation, along with exploring the existing barriers to oncofertility establishment in India.

## Methods

The study was approved by the Institutional Ethics Committee, Kasturba Medical College & Kasturba Hospital, Manipal, India (IEC No: 880/2017), and is in continuation of a previously published study. ^4^ The earlier work included the use of an eighteen-item survey, which was given to oncologists and gynaecologists to explore their attitudes towards fertility preservation. The survey was designed in collaboration with the Oncofertility Consortium, USA. Content validation for the survey was done by experts in the field ^14^ and a pilot study was conducted on a small group of healthcare providers to validate the comprehensibility of the survey questions (unpublished). In our new study, the survey was administered randomly to GPs and PEDs of the country at various national conferences and virtual platforms from May 2019 to September 2020. Written informed consent was taken from all the subjects participating in the survey.

In brief, the survey contained a total of eighteen questions, of which ten were aimed at understanding the participants’ knowledge, attitudes, and referral trends in oncofertility, including their familiarity with existing oncofertility barriers in India, and sought the respondent’s suggestions, if any, for effective use of fertility preservation. The remaining eight questions covered the demographics of the survey respondents and contextual details such as the number of new cancer patients treated in a month and the patient age groups. The questions were of different complexities, including those of a dichotomous nature, assessment tools with grading scales, multiple choice items where only one response could be selected, multiple response items where more than one answer could be selected, and even open-ended questions. During the analysis, the survey responses were assigned numerical values (Yes=1, No=0), and the data was analysed using descriptive statistics calculated with Microsoft Excel. In questions containing grading scales or multiple choice, options such as ‘not aware’, ‘aware but inadequate knowledge’, ‘knowledgeable’, ‘very knowledgeable’, were clubbed as two categories – ‘inadequate knowledge’ and ‘knowledgeable’. For open-ended questions such as suggestions for oncofertility practice, all the responses were considered and grouped based on similarity of the suggestions made. Questions that showed a high variation in responses, such as more than half of the participants not answering, were not included in the analysis.

## Results

Out of 105 survey forms distributed, 23 responses each from general physicians (GPs) and pediatricians (PEDs) were received, with an average response rate of 43.8%. The demographics of the PCPs are shown in Table 1.

**Table 1.** Demographics of primary care physicians (PCPs) participating in the survey.

	General physicians (%) n=23	Pediatricians (%) n=23
**Age groups (years)**		
<30	30.4	21.7
31–40	17.3	21.7
41–50	21.7	34.7
51–60	26	21.7
>60	4.3	0
**Gender**		
Male	86.9	73.9
Female	13	26
**Work experience (years)**		
<5	39.1	26
5–10	8.6	17.3
11–15	8.6	8.6
16–20	4.3	13
>20	39.1	34.7
**Practice setting type**		
Academic institution	43.4	60.8
Government/Aided institution	0	4.3
Private practice affiliated with institution	4.3	21.7
Exclusive private practice	52.1	13

### Awareness of fertility preservation guidelines and options

**Figure 1.**
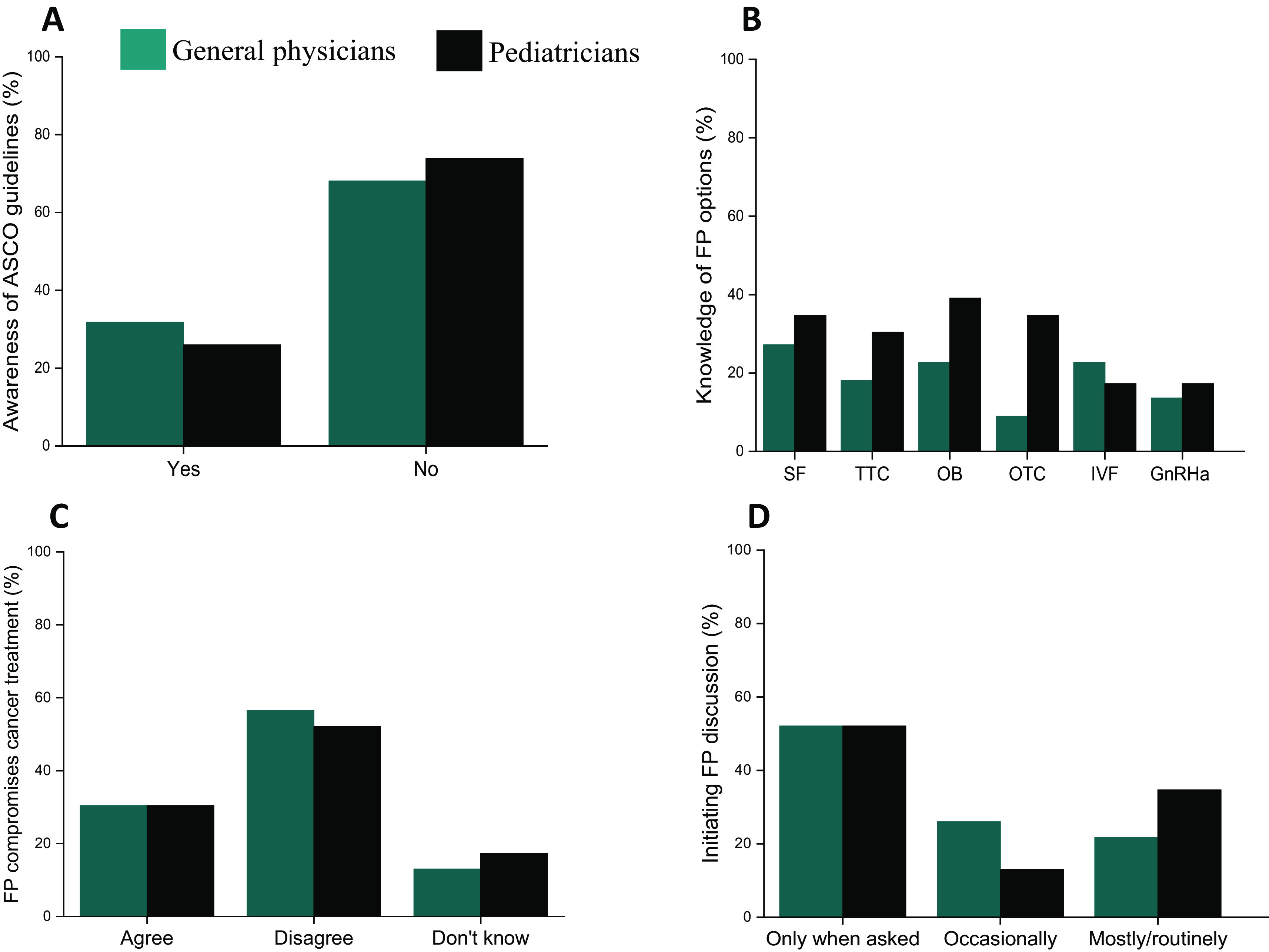
Trends in fertility preservation among primary care physicians (PCPs) – general physicians and pediatricians. (A) Awareness of ASCO fertility preservation guidelines among PCPs. (B) PCPs’ knowledge of various fertility preservation options. The various options on the X axis are: Sperm freezing (SF), Testicular tissue cryopreservation (TTC), Oocyte banking (OB), Ovarian tissue cryopreservation (OTC), IVF followed by embryo freezing (IVF) and GnRHa pre-treatment (GnRHa). (C) Opinion on whether fertility preservation compromises cancer treatment. (D) Frequency of initiating fertility preservation discussions with cancer patients (teal bar: general physicians; black bar: pediatricians).

About 32% of GPs (7 of 22) and 26% of PEDs (6 of 23) were aware of the American Society for Clinical Oncology (ASCO) guidelines on fertility preservation ( Figure 1A). When assessing the knowledge of different fertility preservation options available for both prepubertal and adult cancer patients, about 60% of the PCPs did not have an adequate knowledge on established fertility preservation (FP) options ( Figure 1B). However, PEDs had a slightly higher level of knowledge of some of the FP options such as sperm banking (35%; 8 of 23), immature testicular tissue freezing (30%; 7 of 23), ovarian tissue freezing (35%; 8 of 23) and oocyte freezing (39%; 9 of 23), and were more knowledgeable of the time needed to undertake each of the options compared to GPs.

### Perception towards FP, referral trends

When asked their opinion on the statement, “offering fertility preservation compromises cancer treatment”, 56% of GPs (13 of 23) and 52% of PEDs (12 of 23) disagreed with the statement, indicating a positive attitude towards oncofertility ( Figure 1C). Further, the frequency of fertility preservation counselling or referrals showed that 78% of GPs (18 of 23) and 65% of PEDs (15 of 23) initiated FP discussions with the cancer-diagnosed patients only occasionally or when asked; only 21-35% of them reported routine discussions ( Figure 1D).

### Comfort level in discussing FP

The majority of PCPs (>60%) in both groups were comfortable discussing most of the available FP options. However, when it came to female fertility preservation options, a minority of GPs were not comfortable discussing oocyte freezing (37%; 7 of 19), ovarian tissue freezing (35%; 7 of 17) or IVF (33%; 6 of 18) options, which could be reflective of inadequate knowledge in this area.

### Barriers and suggestions towards oncofertility with reference to the Indian context

**Figure 2.**
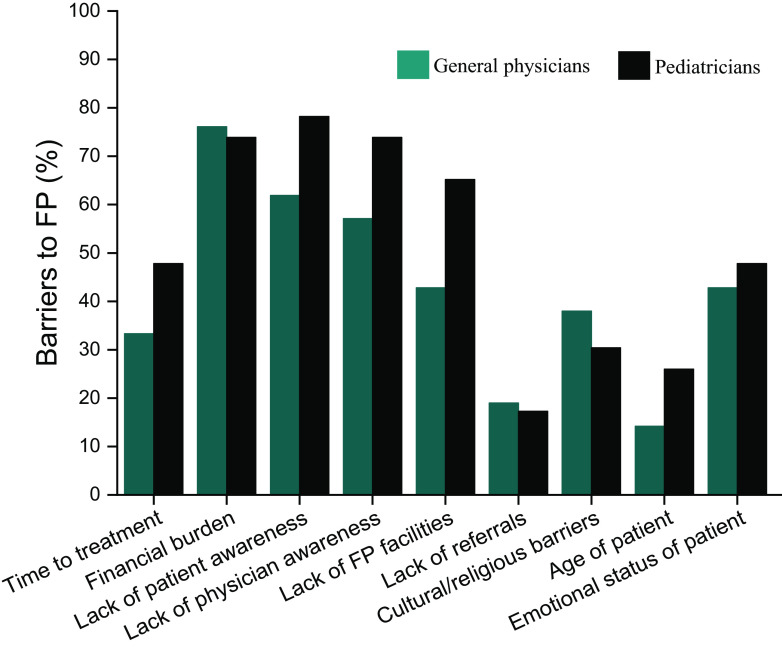
Barriers to effective utilization of fertility preservation services, as perceived by primary care physicians (PCPs). (Teal bar: general physicians; black bar: pediatricians.)

Participants were asked to select the most appropriate of the listed barriers for effective oncofertility implementation in India. PCPs from both groups felt ‘financial burden’ (76% of GPs, 74% of PEDs), ‘lack of patient awareness’ (62% of GPs, 78% of PEDs) and ‘lack of physician awareness’ (57% of GPs, 74% of PEDs) to be the biggest barriers to FP. PCPs felt that ‘lack of FP facilities’ (43% of GPs, 65% of PEDs) and ‘emotional status of patient’ (43% of GPs, 48% of PEDs) were other important barriers ( Figure 2).

One of the major suggestions given, in the open-ended questions, was to create ‘awareness among primary healthcare providers in fertility preservation’ (36% of GPs, 67% of PEDs). More than 40% of GPs and PEDs felt there was a need for bringing about social awareness among the general public about oncofertility programs and educating the patient and their family. Other suggestions included, ‘routine medical education’, and ‘affordable costs’, which would help bring about effective implementation of oncofertility programs.

## Discussion

This study showed, for the first time, the perceptions and attitudes towards oncofertility among PCPs in India. Lack of knowledge about the current international fertility preservation (FP guidelines) and of the available FP options were key observations made in this study.

In India, the established fertility preservation options include sperm, oocyte and embryo banking for post-pubertal and adult cancer patients while prepubertal options that are available include ovarian or immature testicular tissue cryopreservation, which are still considered experimental. Other options such as gonadal shielding, GnRH analogs are also offered to cancer-diagnosed patients, similar to developed countries. ^15^ However, the fertility preservation referral rates are only about 4-12% even in European countries. ^16^ In India, while there are no studies reporting referral rates, the number of patients receiving fertility preservation counselling is quite low. A study reported that only 15% of childhood cancer survivors received information on infertility risks though they were about to receive high-gonadotoxic risk cancer treatment. ^17^ About 87% of patients with newly diagnosed cancers reported a desire to understand the long-term risks resultant of their disease and treatment. ^18^

The majority of PCPs participating in our study reported inadequate knowledge of even the established fertility preservation options along with a reduced awareness of the American Society of Clinical Oncology Fertility Preservation (ASCO-FP) guidelines. This is in line with a cross-sectional study in India on female cancer patients of reproductive age revealing that 68% reported a lack of information received from primary physicians about fertility risks and FP options. ^19^ There could be several contributing factors to the above finding in our study, one of which could be the lack of knowledge about FP in India, since more than 90% of healthcare workers in other parts of the country reported the need for continued medical education programs or seminars in oncofertility. ^20^ Secondly, the ASCO-FP guidelines are mainly targeted to oncologists and other cancer-care providers, ^2^ and not to primary care physicians, though organizations such as the American Academy of Pediatrics have recommended fertility preservation counselling for pediatric and adolescent patients. ^13^ While ASCO has released primary care focused cancer survivorship guidelines for a few cancers, ^12^^,^^21^ there is a need for fertility preservation guidelines before the onset of cancer treatment in pediatric and reproductive-age patients, that can be applicable to primary care settings. Also, PCPs’ depth of knowledge pertaining to long-term effects of cancer treatment could be limited due to their lack of exposure to relevant literature pertaining to the field, ^22^ therefore, conducting educational programs for PCPs to increase awareness and knowledge of the various fertility preservation options could aid PCPs in their discussions with patients. In an online survey conducted by Nahata *et al.*, it was seen that the majority of pediatricians felt inadequately trained in fertility risks and sexual function, which was reflective in their comfort level when discussing such issues. ^23^ Though our study reported a higher comfort level among PEDs in discussing most FP options, a majority of them have suggested the need for fertility preservation awareness programs.

In India, the burden of cancer is steadily increasing, with the Globocan project predicting 1.7 million new cases by 2035. ^24^ Among children of 0-14yrs and children and adolescents of 0-19yrs, approximately 52,366 individuals and 76,805 individuals develop cancer every year, respectively. ^25^ However, the ratio of new cancer cases per clinical oncologist is 677:1 and it has been reported that medical oncologists from low- and middle-income countries such as India have a substantial workload compared to high-income countries. ^26^^–^^28^ While the onus of FP counselling is placed on oncologists and gynecologists, ^2^ in the given situation, an integrated care model involving PCPs would be more appropriate. As PCPs have a continual relationship with the patients and are more familiar with their wishes, PCPs can be a part of the FP referrals or discussions, to enable patients to think through their decisions, rather than these discussions taking place only with the oncologists or surgeons. ^29^^–^^31^ For this to succeed however, knowledge dissemination and awareness building of fertility preservation among PCPs in developing countries like India is essential, as emphasized by the findings of the present study. Our earlier study specifically looked at oncologists and gynaecologists’ attitude towards fertility preservation, who also emphasized the need for oncofertility awareness. ^4^

The scope of oncofertility has started to include fertility preservation in patients with non-oncological conditions who are at high risk of infertility due to gonadotoxic treatments, or individuals with sexual development or auto-immune disorders, and also, in the gender-diverse population. ^32^ The successful establishment of fertility preservation in (non)-oncological conditions therefore requires physicians, both primary care and specialists, to have adequate knowledge of fertility preservation and its related guidelines. A shared care model that facilitates sustained collaborations between healthcare providers, both primary and acute care, will ensure successful execution of fertility preservation goals in patients whose fertility could be impacted. ^1^ Robust strategies including co-ordinated referral pathways, use of tele-health platforms and decision-aids will promote oncofertility referrals by enabling patients to take informed decisions towards preserving their fertility. ^1^ Anazodo *et al.*, identified several themes for delivery of oncofertility care such as age-appropriate referral pathways, collaboration between healthcare providers in pediatric or adult hospitals and fertility clinics, and oncofertility training of both cancer and non-cancer HCPs in order to improve the quality of care. ^1^

Barriers in providing fertility preservation care can lead to their under-implementation. Our study reported financial burden on the patient, as an important barrier to oncofertility by majority of the PCPs. As fertility preservation procedures are not covered under insurance costs in India, ^33^ this is one of the biggest hurdles that need to be overcome. Similar economic barriers have been reported in other developing as well as developed countries. ^33^^,^^34^ Another important barrier reported in the study were lack of fertility preservation awareness among physicians.

This can be overcome by providing educational resources, for e.g., decision aids, brochures or conducting Continuing Medical Education (CME) programs which is expected to increase the knowledge and confidence in healthcare workers thereby improving the frequency as well as quality of fertility preservation discussions. ^35^^,^^36^

Strategies for effective implementation of FP services across the country can include formation of fertility preservation consortiums that allow for knowledge and resource distribution among healthcare providers would facilitate successful establishment of oncofertility care in India. ^37^ Development of clinical guidelines with an objective to provide guidance on the gonadotoxicity of (non)-oncological treatments can be focused towards primary care to bridge the knowledge gaps among PCPs in the country.

## Conclusions

Our pilot study highlights the dearth of oncofertility awareness among PCPs. With increasing involvement of PCPs in the cancer care continuum, this group could play a crucial role in patient decision-making, thereby helping in the successful establishment of oncofertility as part of standard cancer care. As the scope of fertility preservation is expanding to also include non-oncological patients requiring gonadotoxic therapies the involvement of PCPs whom the patients know and trust and who are also familiar with the patient’s wishes or life goals, in fertility preservation discussions could prove beneficial to the cancer-diagnosed patients as well as increase the frequency of fertility preservation referrals. The present study emphasizes the need for knowledge dissemination and awareness about oncofertility among PCPs. This study should contribute to what is currently only limited literature in this area. Limitations of this study include a small sample size and selection bias due to random recruitment of survey participants at conferences. Due to the small sample size conclusions could not be drawn on the characteristics of PCPs with increased knowledge on fertility preservation.

## Author contributions

PT: Collected and analyzed the data, wrote paper; SU, RKJ, KSU, GK, NS, TKW: Revised the manuscript critically for important intellectual content; SKA: Conceived and designed the study, wrote paper. PT is the guarantor of this work and as such, had full access to all the data and takes responsibility for the integrity of the data and the accuracy of the data analysis. All authors have given final approval for publication.

## Data Availability

Open Science Framework: ‘Oncofertility awareness among primary care physicians in India.’ https://doi.org/10.17605/OSF.IO/N7GVKh. ^12^ This project contains the following underlying data:
- Oncofertility survey, data description sheet and content validity analysis (zip file) - Survey responses (raw data) (zip file) Data are available under the terms of the Creative Commons Zero “No rights reserved” data waiver (CC0 1.0 Public domain dedication).
