# Supplementary material for: Oncofertility awareness among primary care physicians in India
Source: F1000Res. 2023 Sep 28;12:153. Originally published 2023 Feb 10. [Version 2] doi: 10.12688/f1000research.126232.2 (PMC10521121; doi:10.12688/f1000research.126232.2)
Supplement: Peer review history

# Reviewer report: Reviewer response for version 2

**DOI:** 10.5256/f1000research.156275.r229233

**Published:** 2024-01-04

**Authors:** Giuliano Bedoschi

**Affiliations:** 1 Department of Gynecology and Obstetrics, University of Sao Paulo, Ribeirão Preto, São Paulo, Brazil

Dear authors,

 Congratulations on your manuscript titled "Oncofertility awareness among primary care physicians in India" Your work goes a long way in mapping the knowledge in this important area by linking oncofertility with the role of primary care physicians (PCPs) in India.

 Your manuscript first highlights the importance of integrating cancer treatment and fertility preservation and emphasizes the importance of oncofertility as part of standard care for reproductive-age cancer patients and prepubertal children undergoing gonadotoxic therapies. The role of general practitioners, including general practitioners (GPs) and pediatricians (PEDs), is highlighted as crucial in the referral system for the successful implementation of oncofertility. The expanded role of GPs in cancer care, from prevention to survivorship, is highlighted, as is the need to include them in fertility preservation discussions and referrals.

 The Methods section describes the process of interviewing PCPs and exploring their awareness, knowledge, and attitudes towards FP. The ethical approval of the study and the design and validation of the survey contribute to its credibility.

 The results section details the data on awareness of FP guidelines and options among PCPs. Particularly insightful are the identified barriers to effective implementation of oncofertility, such as financial burdens and lack of awareness.

 In the discussion, address the perceptions and attitudes of PCPs in India towards oncofertility and highlight the lack of knowledge about fertility preservation options and guidelines. The need for guidelines and education programs that focus on fertility preservation is well argued. The increasing burden of cancer in India and the important role of PCPs in the referral process are effectively highlighted.

 Although the study design is commendable, the methods are well described and the discussion is adequate, I recommend that one important addition be made: the inclusion of the study limitations in the discussion section. This would provide a more comprehensive understanding of the context and potential limitations of your findings.

 I also suggest adding the discussion of the challenges of oncofertility programs, particularly in resource-limited settings (1). This discussion may provide additional insights into the global context of oncofertility that are particularly relevant to the Indian scenario described in your manuscript.

 We thank you for your valuable contribution to this topic. Your research is instrumental in improving our understanding of oncofertility and the important role of family physicians in this area.

 Sincerely,

Is the work clearly and accurately presented and does it cite the current literature?

Yes

If applicable, is the statistical analysis and its interpretation appropriate?

Yes

Are all the source data underlying the results available to ensure full reproducibility?

Yes

Is the study design appropriate and is the work technically sound?

Yes

Are the conclusions drawn adequately supported by the results?

Yes

Are sufficient details of methods and analysis provided to allow replication by others?

Yes

Reviewer Expertise:

Fertility Preservation

I confirm that I have read this submission and believe that I have an appropriate level of expertise to confirm that it is of an acceptable scientific standard, however I have significant reservations, as outlined above.

## References

[1] : Oncofertility programs still suffer from insufficient resources in limited settings. *Journal of Assisted Reproduction and Genetics* .2022;39(4) : 10.1007/s10815-022-02452-w 953-955 10.1007/s10815-022-02452-w PMC905097235257262

# Reviewer report: Reviewer response for version 1

**DOI:** 10.5256/f1000research.138622.r178269

**Published:** 2023-09-14

**Authors:** Alex C. Varghese

**Affiliations:** 1 Fertility Associates, Auckland, New Zealand

The manuscript entitled "Oncofertility awareness among primary care physicians in India" provides a preliminary survey based analysis highlighting the dearth of oncofertility awareness among PCPs. Specific comments are:

 1) The discussion section can have the following additional details: 

- Any published estimates of reproductive cancers/prepubertal cancers in India per population/incidences.
- A paragraph on the current status of fertility preservation in IVF units across the country and gaps in services. Anazodo *et al.* (2019 ^1^) described a model to increase the oncofertility implementation - worth mentioning in the current manuscript. 
- The authors have mentioned about the need for the shared care model for oncofertility referrals. Please explain how this could be achieved in an Indian context tailored to the identified determinants and how the improvement strategies for the guideline-specific barriers should be developed.

Is the work clearly and accurately presented and does it cite the current literature?

Yes

If applicable, is the statistical analysis and its interpretation appropriate?

Yes

Are all the source data underlying the results available to ensure full reproducibility?

Yes

Is the study design appropriate and is the work technically sound?

Yes

Are the conclusions drawn adequately supported by the results?

Yes

Are sufficient details of methods and analysis provided to allow replication by others?

Yes

Reviewer Expertise:

Assisted Reproductive Technologies

I confirm that I have read this submission and believe that I have an appropriate level of expertise to confirm that it is of an acceptable scientific standard.

## References

[1] : How can we improve oncofertility care for patients? A systematic scoping review of current international practice and models of care. *Hum Reprod Update* .2019;25(2) : 10.1093/humupd/dmy038 159-179 10.1093/humupd/dmy038 30462263 PMC6390168

# Response

**Published:** 2023-09-18

**Authors:** Satish Adiga

**Affiliations:** Manipal Academy of Higher Education, India

We thank the reviewer for the constructive comments. The manuscript has been revised accordingly. Hereby addressing the reviewer’s comments:

 1) The discussion section can have the following additional details:

 Any published estimates of reproductive cancers/prepubertal cancers in India per population/incidences.

Ans: Thank you for the suggestion, it has now been added accordingly.

 2. A paragraph on the current status of fertility preservation in IVF units across the country and gaps in services. Anazodo et al. (20191) described a model to increase the oncofertility implementation - worth mentioning in the current manuscript.

Ans: Thank you for the suggestion. There is however, currently no data reporting the fertility preservation referral rates in India, but there are a few studies reporting the frequency of counselling for infertility-risk, which has now been added.

 The models of care mentioned in the recommended publication have now been added in the discussion section.

 3. The authors have mentioned about the need for the shared care model for oncofertility referrals. Please explain how this could be achieved in an Indian context tailored to the identified determinants and how the improvement strategies for the guideline-specific barriers should be developed.

Ans: Thank you for the suggestion. It has been addressed to the best of the author’s knowledge and available literature which is limited.

# Reviewer report: Reviewer response for version 1

**DOI:** 10.5256/f1000research.138622.r190272

**Published:** 2023-08-25

**Authors:** Regina Barragan-Carrillo

**Affiliations:** 1 Department of Hematology and Oncology, Instituto Nacional de Ciencias Médicas y Nutrición "Salvador Zubirán", Mexico City, Mexico

I reviewed thoughtfully the manuscript Oncofertility awareness among primary care physicians in India. In this the authors present an exploration of the current knowledge and barriers to fertility preservation in India among PCPs. I congratulate the authors for addressing a topic needing assessment in LMICs.

 My main concerns are regarding the paper's impact in the current knowledge, as usually young patients diagnosed with a malignancy are under the care of a specialized team (pediatric oncologist/hematologist), and I do not fully understand if the lack of knowledge of PCP in such a specialized topic is something of importance in their care-continuum. I think if the authors could really justify the importance of understanding the knowledge of PCP in their paper, it could strengthen it.

 Overall, I found the paper well-written and understandable.

 The data presented was only a description of the obtained answers, were there any correlations made to understand? For example, what are the characteristics of the physicians with the highest knowledge of oncofertility?

 During the discussion, there have been other publications in LMICs for example, in Mexico, also in other parts of the world outside the US like Italy. I recommend making a more exhaustive review of the currently published literature to correlate your results with the currently available literature.

 During the discussion I would also recommend a deeper exploration of the reported barriers, are these similar to their context? Or as these unique in the Indian population?

 In the conclusion, once again I would clearly state why you consider that this is relevant information for the reader.

Is the work clearly and accurately presented and does it cite the current literature?

Partly

If applicable, is the statistical analysis and its interpretation appropriate?

No

Are all the source data underlying the results available to ensure full reproducibility?

No

Is the study design appropriate and is the work technically sound?

Partly

Are the conclusions drawn adequately supported by the results?

No

Are sufficient details of methods and analysis provided to allow replication by others?

No

Reviewer Expertise:

Urologic oncology, disparities in cancer care

I confirm that I have read this submission and believe that I have an appropriate level of expertise to state that I do not consider it to be of an acceptable scientific standard, for reasons outlined above.

# Response

**Published:** 2023-09-18

**Authors:** Satish Adiga

**Affiliations:** Manipal Academy of Higher Education, India

We thank the reviewer for the constructive comments. The manuscript has been revised accordingly. Hereby addressing the reviewer’s comments:

 1. My main concerns are regarding the paper's impact in the current knowledge, as usually young patients diagnosed with a malignancy are under the care of a specialized team (pediatric oncologist/hematologist), and I do not fully understand if the lack of knowledge of PCP in such a specialized topic is something of importance in their care-continuum. I think if the authors could really justify the importance of understanding the knowledge of PCP in their paper, it could strengthen it.

Ans: We agree with the reviewer’s concern. The cancer patients are usually referred to specialized team, but literature as well as discussions with PCPs during the survey distribution have shown that these patients interact with their PCPs during the course of cancer treatment and wish to have their PCPs involved. The introduction section has been revised to emphasize further the role of PCPs in the cancer care continuum.

 2. The data presented was only a description of the obtained answers, were there any correlations made to understand? For example, what are the characteristics of the physicians with the highest knowledge of oncofertility?

Ans: Due to the small sample size in each group conclusions could not be drawn regarding the characteristics of the PCPs with higher oncofertility knowledge. This has now been mentioned as a limitation of the study.

 3. During the discussion, there have been other publications in LMICs for example, in Mexico, also in other parts of the world outside the US like Italy. I recommend making a more exhaustive review of the currently published literature to correlate your results with the currently available literature. During the discussion I would also recommend a deeper exploration of the reported barriers, are these similar to their context? Or as these unique in the Indian population?

Ans: Thank you for the suggestion. We agree, there have been other publications about oncofertility trends among oncologists or gynaecologists. However, among PCPs there is very limited literature, and this manuscript is the first of its kind, especially in India. Oncofertility awareness among PCPs is an area with great potential to leverage the role of PCPs in successful oncofertility referrals. Hence, the limited review based on existing literature in this area. However, as per the reviewer’s suggestions, the barriers have been compared with other developing countries and revised accordingly.

 4. In the conclusion, once again I would clearly state why you consider that this is relevant information for the reader.

Ans: Thank you for the suggestion. It has now been addressed and revised accordingly.

# Reviewer report: Reviewer response for version 1

**DOI:** 10.5256/f1000research.138622.r178270

**Published:** 2023-07-17

**Authors:** Padma Rekha Jirge

**Affiliations:** 1 Department of Reproductive Medicine, Shreyas Hospital and Sushrut Assisted Conception Clinic, Kolhapur, India

It is a well conducted and well written manuscript. It addresses a relevant issue. It is interesting that the authors have gone beyond the specialist and interviewed primary care physicians. It is an important step towards improving awareness about oncofertility. The limitations of the study are the small numbers included.

Is the work clearly and accurately presented and does it cite the current literature?

Yes

If applicable, is the statistical analysis and its interpretation appropriate?

Yes

Are all the source data underlying the results available to ensure full reproducibility?

Yes

Is the study design appropriate and is the work technically sound?

Yes

Are the conclusions drawn adequately supported by the results?

Yes

Are sufficient details of methods and analysis provided to allow replication by others?

Yes

Reviewer Expertise:

Ovarian reserve

I confirm that I have read this submission and believe that I have an appropriate level of expertise to confirm that it is of an acceptable scientific standard.

# Response

**Published:** 2023-09-18

**Authors:** Satish Adiga

**Affiliations:** Manipal Academy of Higher Education, India

We thank the reviewer for the constructive comments.
